# A phase I open-label dose-escalation study of the anti-HER3 monoclonal antibody LJM716 in patients with advanced squamous cell carcinoma of the esophagus or head and neck and HER2-overexpressing breast or gastric cancer

**DOI:** 10.1186/s12885-017-3641-6

**Published:** 2017-09-12

**Authors:** Kerry Lynn Reynolds, Philippe L. Bedard, Se-Hoon Lee, Chia-Chi Lin, Josep Tabernero, Maria Alsina, Ezra Cohen, José Baselga, George Blumenschein, Donna M. Graham, Ignacio Garrido-Laguna, Dejan Juric, Sunil Sharma, Ravi Salgia, Abdelkader Seroutou, Xianbin Tian, Rose Fernandez, Alex Morozov, Qing Sheng, Thiruvamoor Ramkumar, Angela Zubel, Yung-Jue Bang

**Affiliations:** 1 Massachusetts General Hospital, 55 Fruit Street, Boston, MA 02114 USA; 2 Princess Margaret Cancer Centre, Toronto, ON Canada; 3 Seoul National University College of Medicine, Seoul, Republic of Korea; 4 National Taiwan University Hospital, Taipei, Taiwan; 5 Vall d’Hebron University Hospital and Institute of Oncology (VHIO), Universitat Autònoma de Barcelona, Barcelona, Spain; 6 Moores Cancer Center, University of California at San Diego, La Jolla, CA USA; 7 Memorial Sloan Kettering Cancer Center, New York, NY USA; 8 Department of Thoracic/Head and Neck Medical Oncology, The University of Texas MD Anderson Cancer Center, Houston, TX USA; 9 Huntsman Cancer Institute, Salt Lake City, UT USA; 10 University of Chicago, Chicago, IL USA; 11 Novartis Pharma AG, Basel, Switzerland; 12 Novartis Pharmaceuticals Corporation, East Hanover, NJ USA; 13 Novartis Institutes for BioMedical Research, Cambridge, MA USA; 14 City of Hope, Department of Medical Oncology and Therapeutics Research, Duarte, CA USA; 15 Pfizer Inc., New York, NY USA

**Keywords:** Phase I, HER3, HER2, Monoclonal antibody, LJM716

## Abstract

**Background:**

Human epidermal growth factor receptor 3 (HER3) is important in maintaining epidermal growth factor receptor-driven cancers and mediating resistance to targeted therapy. A phase I study of anti-HER3 monoclonal antibody LJM716 was conducted with the primary objective to identify the maximum tolerated dose (MTD) and/or recommended dose for expansion (RDE), and dosing schedule. Secondary objectives were to characterize safety/tolerability, pharmacokinetics, pharmacodynamics, and preliminary antitumor activity.

**Methods:**

This open-label, dose-finding study comprised dose escalation, followed by expansion in patients with squamous cell carcinoma of the head and neck or esophagus, and HER2-overexpressing metastatic breast cancer or gastric cancer. During dose escalation, patients received LJM716 intravenous once weekly (QW) or every two weeks (Q2W), in 28-day cycles. An adaptive Bayesian logistic regression model was used to guide dose escalation and establish the RDE. Exploratory pharmacodynamic tumor studies evaluated modulation of HER3 signaling.

**Results:**

Patients received LJM716 3–40 mg/kg QW and 20 mg/kg Q2W (54 patients; 36 patients at 40 mg/kg QW). No dose-limiting toxicities (DLTs) were reported during dose-escalation. One patient experienced two DLTs (diarrhea, hypokalemia [both grade 3]) in the expansion phase. The RDE was 40 mg/kg QW, providing drug levels above the preclinical minimum effective concentration. One patient with gastric cancer had an unconfirmed partial response; 17/54 patients had stable disease, two lasting >30 weeks. Down-modulation of phospho-HER3 was observed in paired tumor samples.

**Conclusions:**

LJM716 was well tolerated; the MTD was not reached, and the RDE was 40 mg/kg QW. Further development of LJM716 is ongoing.

**Trial registration:**

Clinicaltrials.gov registry number NCT01598077 (registered on 4 May, 2012).

**Electronic supplementary material:**

The online version of this article (10.1186/s12885-017-3641-6) contains supplementary material, which is available to authorized users.

## Background

The receptor tyrosine-protein kinase (RTK) of the v-erb-b2 erythroblastic leukemia viral oncogene homolog (ErbB) receptor tyrosine kinase family, ErbB-3 or human epidermal growth factor receptor 3 (HER3), is implicated in tumor growth, proliferation, chemotherapeutic resistance, and the promotion of invasion and metastasis [1]. The HER3 protein lacks significant kinase activity and is activated through heterodimerization with other RTKs such as epidermal growth factor receptor (EGFR) and human epidermal growth factor receptor 2 (HER2) [2], and HER3 is the preferred dimerization partner of the latter [3]. Dimerization can result from either overexpression of HER2, in a ligand-independent manner, or through receptor-mediated activation by the ligand neuregulin 1 (NRG1; also known as heregulin), and this leads to HER2:HER3-mediated oncogenic activation of phosphoinositide 3-kinase (PI3K) signaling [4]. HER3 thus plays an important role in maintaining EGFR- and HER2-driven cancers and mediating resistance to EGFR- and HER2-targeted therapy [5].

HER2:HER3-directed therapies benefit patients with HER-overexpressing tumors. For example, a significant improvement in overall survival was reported for the combination of the HER2 dimerization-inhibiting antibody pertuzumab with trastuzumab and docetaxel in patients with HER2-positive metastatic breast cancer [6]. Potential target HER2-overexpressing tumors include esophageal squamous cell carcinoma (ESCC; 31% HER2 positive) [7], metastatic breast cancer (20–30% HER2 positive) [8], and metastatic gastric/gastroesophageal junction cancer (16% HER2 positive) [9]. Preclinical data indicate that NRG1-driven tumors, including those lacking *HER2* amplification, may also respond to HER2:HER3-directed therapy [4]. NRG1-mediated autocrine signaling has been documented in a significant subset of head and neck tumors [10], and NRG1 expression is particularly enriched in squamous cell carcinoma of the head and neck (SCCHN) [11]. Squamous cell carcinomas in general have been found to exhibit relatively high NRG1 expression compared with adenocarcinoma counterparts [12]. NRG1 expression is also a predictive biomarker for response to anti-HER3 therapy in human tumor xenograft models, including breast, head and neck, and esophageal cancers [11].

LJM716 is a fully human anti-HER3 immunoglobulin G1 (IgG1) monoclonal antibody. LJM716 is distinct from other HER2:HER3-targeted therapies in that it binds a conformational epitope that traps HER3 in the inactive conformation preventing its receptor activation, and possesses the unique ability to inhibit both ligand-induced and ligand-independent activation of HER3. Both mechanisms of HER3 activation can be targeted in a number of different tumor types by LJM716, which displays single-agent antitumor activity in a range of HER2-amplified and NRG1-expressing xenograft models [4]. Here, we evaluate the safety and tolerability of single-agent LJM716 in patients with HER2-positive breast cancer or gastric cancer, or with ESCC or SCCHN regardless of HER2 status.

## Methods

### Study oversight

This open-label, multicenter phase I study (clinicaltrials.gov registry number NCT01598077) was conducted at eight clinical centers across five countries (USA, Canada, Spain, Republic of Korea, and Taiwan). The accrual period was from July 26, 2012 to March 13, 2014. This study was performed in accordance with the Declaration of Helsinki and the principles of Good Clinical Practice. The protocol was approved by an Institutional Review Board at each hospital, and all patients provided written informed consent before any study procedures. The study was designed by the sponsor (Novartis Pharmaceuticals Corporation). The sponsor collected the data and analyzed them in conjunction with the authors.

### Patient selection

All included patients fulfilled the following inclusion criteria: male or female aged ≥18 years, Eastern Cooperative Oncology Group (ECOG) performance score ≤ 2, HER2-positive locally advanced/metastatic breast cancer or gastric cancer, recurrent or metastatic SCCHN or ESCC regardless of HER2 status, with no other available effective treatment option existing (investigator decision). For breast cancer, patients were required to have documented HER2 overexpression by immunohistochemistry (IHC) 3+ or amplification by in situ hybridization according to standard guidelines [13, 14]. For gastric cancer (including gastroesophageal junction tumors), patients were required to have documented HER2 overexpression as IHC 3+ or IHC 2+ with amplification by in situ hybridization [13, 14]. For the dose-expansion phase of the study, patients (with no pre-specified tumor type) were required to have a baseline tumor biopsy and measurable disease as defined by Response Evaluation Criteria in Solid Tumors (RECIST) version 1.1 [15]. There was no limit on the number of prior antineoplastic regimens received. Eligible patients had adequate hepatic, renal, and hematologic functions. Exclusion criteria included patients with untreated and/or symptomatic central nervous system metastases, impaired cardiac function, a history of another primary malignancy requiring treatment, and if they had received prior anti-HER3 antibody treatment.

### Study objectives

The primary objectives of the study were to determine the maximum tolerated dose (MTD) and/or recommended dose for expansion (RDE) and preferred dosing schedule of LJM716 as a single agent when administered intravenously (IV) to adult patients with SCCHN, or ESCC, or HER2-positive metastatic breast cancer or gastric cancer. Secondary objectives were to characterize the safety and tolerability of LJM716, to characterize the pharmacokinetics and pharmacodynamic response in tumor tissue, including the relationship between tumor HER3 inhibition and suppression of downstream signaling, to assess the preliminary antitumor activity (overall response rate [ORR], progression-free survival [PFS], and duration of response) of LJM716, and to assess the emergence of antibodies against LJM716.

### Study design and treatment plan

The study consisted of a dose-escalation and a dose-expansion phase. The MTD/RDE had to be established in the dose-escalation phase from a minimum of 15 treated patients. Once established, further patients were recruited and treated at the MTD/RDE in the expansion phase to further evaluate safety, tolerability, and the antitumor activity of LJM716. The study design considered four doses of LJM716 and two administration schedules in the dose-escalation phase, starting at 3 mg/kg; then 10, 20, and 40 mg/kg once weekly (QW), and 20 mg/kg or 40 mg/kg every 2 weeks (Q2W) of a 28-day treatment cycle. LJM716 was administered by intravenous infusion over 2 h. As a result of infusion-related reactions (IRRs) during ongoing clinical studies on LJM716, a premedication regimen of acetaminophen 650 mg or equivalent, and diphenhydramine 50 mg IV or equivalent, was recommended to prevent IRR development. Each patient was allowed only one dose reduction or a dose interruption ≤28 days in case of toxicity. LJM716 administration was discontinued in patients who had disease progression or experienced a dose-limiting toxicity (DLT) or other unacceptable toxicity, at the discretion of the investigator, or by patient withdrawal.

### Statistical analysis

An adaptive Bayesian logistic regression model (BLRM) [16] incorporating escalation with overdose control (EWOC) criteria was used to guide dose-escalation decisions [17, 18] and establish the MTD and/or RDE for LJM716. The two-parameter BLRM used for dose escalation included covariates to allow for changes to dosing schedule. The model was of the form

$$ Logit\left({\pi}_d\right)=\mathit{\log}\left(\alpha \right)+\beta \bullet \mathit{\log}\left(\frac{d}{d^{\ast }}\right)+{\gamma}_1\bullet {I}_{\left(Q2W/Q4W\right)}+{\gamma}_2\bullet {I}_{Q4W} $$

where *d* represents the total Cycle 1 dose, d* represents the reference dose of 40 mg/kg, π_d_ is the probability of a DLT at dose *d*, and *I*_*(Q2W/Q4W)*_ and *I*_*Q4W*_ are indicator variables that take the value 1 if administration is once every 2 or 4 weeks (*I*_*(Q2W/Q4W)*_) or every 4 weeks (*I*_*Q4W*_). The probability of a DLT at the reference dose under QW administration is therefore represented by*α*, while *γ*_*1*_ and *γ*_*2*_ represent the expected log-odds ratios for the probability of a DLT between the Q2W and QW schedules, and the Q4W and Q2W schedules, respectively.

The Kaplan–Meier method was used to estimate median PFS, and estimated PFS rates at fixed time points. These statistics were provided as point estimates with 95% confidence intervals (CI) if appropriate.

### Toxicity assessments

Safety assessments were carried out based on frequency and severity of all adverse events (AEs) and serious AEs (SAEs), and their relationship to study drug treatment, with regular monitoring of hematology, coagulation, clinical chemistry, pregnancy and urine analysis, performance status, cardiac assessments, vital signs, physical condition, and body weight. Toxicity was graded according to the National Cancer Institute Common Terminology Criteria for Adverse Events, version 4.03 [19]. A DLT was defined as the occurrence of a clinically relevant drug-related AE or abnormal laboratory value assessed as unrelated to disease progression, intercurrent illness, or concomitant medications, and occurring ≤28 days following the first dose in Cycle 1 (see Additional file 1: Table S1 for further details).

### Response assessments

Tumor lesions were assessed as per RECIST v1.1 [15] by study investigators. Patients underwent screening computed tomography (CT) scans of the chest, abdomen, and pelvis, with magnetic resonance imaging (MRI) evaluation of disease not adequately imaged by CT. Tumor assessments were carried out at screening, every two cycles, and at the end of treatment if a scan was not completed within 30 days prior to the end of treatment.

### Pharmacokinetics, pharmacodynamics and exploratory biomarkers, and immunogenicity

For QW dosing in the dose-escalation phase of the study, serum was collected for LJM716 pharmacokinetic assessments during Cycle 1 and at the anticipated steady state (Cycle 3), at pre-infusion, 2, 4, 10, 48, 72, 96, and 168 h post-infusion. Trough samples were collected during dose escalation, for up to 10 cycles. During dose expansion, sparse pharmacokinetic samples were collected at pre-infusion, 2, 4, and 168 h post-infusion in Cycles 1 and 3, and pre-infusion samples were taken once every other cycle for up to 10 cycles. For Q2W dosing, additional time points at 216, 264, and 336 h post-infusion were added to the collection schedule during dose escalation, and were taken pre-infusion, 2, 4, 168, and 336 h post-infusion during dose expansion. Free serum LJM716 concentration was measured using a validated enzyme-linked immunosorbent assay. Parameters determined by a non-compartmental method included, if appropriate, C_max_, T_max_, AUC_0-last_, AUC_0-inf_, CL, V, and T_1/2_. In selected patients during dose escalation, and during dose expansion, paired pre- and post-treatment tumor biopsies were collected for pharmacodynamics studies. Biopsies were taken pretreatment and after 8 weeks of therapy, were snap-frozen and analyzed for levels of total HER3 (t-HER3), and phospho-HER3 (p-HER3) on the Collaborative Enzyme Enhanced Reactive (CEER) immunoassay platform (Prometheus Laboratories Inc., San Diego, CA). *NRG1* RNA was analyzed in archival biopsy specimens by reverse transcription polymerase chain reaction (RT-PCR), to assess tumor *NRG1* gene expression levels as a potential exploratory biomarker that may correlate with efficacy. Next-generation sequencing (NGS) data were also generated on a panel of genes in archival tumor samples (Foundation Medicine, Inc., Cambridge, MA), including baseline mutational status and copy number of the *PIK3CA* gene, and phosphatase and tensin homolog (*PTEN*), in order to evaluate PI3K pathway activation in patient tumors. Serum anti-LJM716 antibody immunogenicity was assessed in each patient at multiple time points, at a minimum during the first cycle and at the end of treatment.

## Results

### Patient characteristics

Between July 26, 2012 and March 13, 2014 (data cut-off date June 27, 2014) a total of 54 patients (24 patients in the dose-escalation phase, and 30 patients in the expansion phase) were treated with LJM716 at doses of 3 mg/kg, 10 mg/kg, 20 mg/kg, and 40 mg/kg QW, and LJM716 at 20 mg/kg Q2W. The median age of patients was 58 years (range 36–78), 38/54 (70%) of patients were aged <65 years, 37/54 (69%) were male, 35/54 (65%) were Caucasian, 40/54 (74%) had an ECOG performance score of 1, and 3/54 (6%) had an ECOG performance score of 2. Patients were heavily pretreated; 30/54 (56%) had received ≥3 prior antineoplastic regimens. Initial diagnoses were SCCHN (*n* = 21 [39%]), ESCC (*n* = 15 [28%]), HER2-overexpressing breast cancer (*n* = 10 [19%]), and gastric cancer (*n* = 8 [15%]); most patients (91%) had ≥ stage IV disease at study entry (Table 1). The median duration of exposure was 8 weeks (range 1.0–47.0 weeks) across all LJM716 doses, and 8 weeks (range 1.0–39.0 weeks) at the RDE; most patients (80%) had an exposure of >4 weeks. All patients discontinued treatment, with disease progression (45 patients [83%]) as the major reason.

**Table 1:** Patient demographics and disease characteristics, by treatment group

	3 mg/kg QW *n* = 1	10 mg/kg QW *n* = 5	20 mg/kg QW *n* = 6	40 mg/kg QW RDE, *n* = 36	20 mg/kg Q2W *n* = 6	All patients *N* = 54
Age, years						
Median (range)	57.0 (57.0–57.0)	55.0 (48.0–66.0)	56.5 (43.0–69.0)	60.5 (36.0–77.0)	54.5 (49.0–78.0)	58.0 (36.0–78.0)
<65 years, *n* (%)	1 (100)	3 (60)	5 (83)	24 (67)	5 (83)	38 (70)
≥65 years, *n* (%)	0	2 (40)	1 (17)	12 (33)	1 (17)	16 (30)
Sex, *n* (%)						
Female	0	2 (40)	2 (33)	11 (31)	2 (33)	17 (31)
Male	1 (100)	3 (60)	4 (67)	25 (69)	4 (67)	37 (69)
Race, *n* (%)						
Asian	0	0	0	17 (47)	0	17 (31)
Black	0	0	0	1 (3)	0	1 (2)
Caucasian	1 (100)	5 (100)	6 (100)	18 (50)	5 (83)	35 (65)
Other	0	0	0	0	1 (17)	1 (2)
ECOG PS, *n* (%)						
0	0	1 (20)	1 (17)	7 (19)	2 (33)	11 (20)
1	1 (100)	4 (80)	5 (83)	26 (72)	4 (67)	40 (74)
2	0	0	0	3 (8)	0	3 (6)
Initial diagnosis, *n* (%)						
Head and neck	1 (100)	4 (80)	2 (33)	12 (33)	2 (33)	21 (39)
Esophageal	0	0	1 (17)	12 (33)	2 (33)	15 (28)
Breast	0	1 (20)	2 (33)	5 (14)	2 (33)	10 (19)
Gastric	0	0	1 (17)	7 (19)	0	8 (15)
Primary tumor histology, *n* (%)						
Squamous cell carcinoma	1 (100)	4 (80)	2 (33)	22 (61)	2 (33)	31 (57)
Other	0	1 (20)	4 (67)	14 (39)	4 (67)	23 (43)
Stage at study entry, *n* (%)						
III–IIIC	0	1 (20)	0	4 (11)	0	5 (9)
IV–IVB	1 (100)	4 (80)	6 (100)	32 (89)	6 (100)	49 (91)
Prior antineoplastic regimen(s), *n* (%)						
No	0	0	0	1 (3)	0	1 (2)
Yes	1 (100)	5 (100)	6 (100)	35 (97)	6 (100)	53 (98)
Prior EGFR or HER2-directed therapy, *n* (%)						
Cetuximab	1 (100)	3 (60)	2 (33)	8 (22)	2 (33)	16 (30)
Pertuzumab	0	0	0	1 (3)	0	1 (2)
Trastuzumab	0	1 (20)	4 (67)	11 (31)	2 (33)	18 (33)
Trastuzumab emtansine	0	1 (20)	0	1 (3)	1 (17)	3 (6)
Number of prior regimens, median (range)	5.0 (5.0–5.0)	4.0 (2.0–7.0)	2.5 (2.0–5.0)	3.0 (1.0–12.0)	3.0 (1.0–4.0)	3.0 (1.0–12.0)

*ECOG* Eastern Cooperative Oncology Group, *EGFR* epidermal growth factor receptor, *HER2* human epidermal growth factor receptor 2

*PS* performance status, *Q2W* once every two weeks, *QW* once weekly, *RDE* recommended dose for expansion

### Dose escalation and toxicity

After each cohort completed Cycle 1 (28 days), the dose chosen was among doses that satisfied both the EWOC and the dose-escalation scheme for LJM716, where maximum increments of up to 0.5 log_10_ for the first escalation (3 mg/kg to 10 mg/kg) and up to 100% thereafter (10 to 20 to 40 mg/kg) were allowed.

No DLTs were reported in the dose-escalation phase of the study. Only one patient had DLTs (grade 3 diarrhea, and grade 3 hypokalemia) during the first cycle of treatment in the expansion phase (40 mg/kg QW). The MTD was not reached and the RDE for the expansion phase was established at 40 mg/kg QW based on the BLRM, applying the EWOC principle and available clinical data including pharmacokinetics, pharmacodynamics, efficacy, and biomarkers during the dose-escalation phase. All patients had at least one AE regardless of study drug relationship. Overall, the most frequent AEs were diarrhea (52%), decreased appetite (44%), pyrexia (41%), fatigue (35%), nausea (35%), IRR (31%), vomiting (30%), constipation and dyspnea (28% each), and anemia and hypomagnesemia (26% each) (Table 2). Grade 3 or 4 AEs, regardless of study drug relationship, occurred in 42 patients (78%) overall. The most frequent grade 3 or 4 AEs were anemia (13%), pneumonia (11%), hypophosphatemia (9%), hypokalemia and dyspnea (7% each), and diarrhea, vomiting, dehydration, pleural effusion, and asthenia (6% each). Overall, 17 patients (31%) reported IRR symptoms as AEs; symptoms were chills (19%), pyrexia (7%), tremor (6%), increased heart rate (4%), and back pain, increased blood pressure, flushing, hypotension, sinus tachycardia, and vomiting (2% each). The most common (≥25%) study drug-related AEs were diarrhea (39%) and IRR (31%) (see Additional file 2: Table S2). Overall, four patients (7%) had grade 3 or 4 AEs suspected to be study drug-related, most frequently diarrhea (4%).

**Table 2:** Adverse events (all grades [≥10%] and grades 3/4, regardless of causality) by treatment group

Preferred term, *n* (%)	3 mg/kg QW *n* = 1		10 mg/kg QW *n* = 5		20 mg/kg QW *n* = 6		40 mg/kg QW RDE, *n* = 36		20 mg/kg Q2W *n* = 6		All patients *N* = 54	
	All grades	Grade 3/4	All grades	Grade 3/4	All grades	Grade 3/4	All grades	Grade 3/4	All grades	Grade 3/4	All grades	Grade 3/4
Diarrhea	0	0	2 (40)	0	5 (83)	1 (17)	18 (50)	1 (3)	3 (50)	1 (17)	28 (52)	3 (6)
Decreased appetite	0	0	3 (60)	0	4 (67)	0	16 (44)	0	1 (17)	0	24 (44)	0
Pyrexia	0	0	2 (40)	0	2 (33)	0	18 (50)	1 (3)	0	0	22 (41)	1 (2)
Fatigue	0	0	2 (40)	0	2 (33)	1 (17)	14 (39)	0	1 (17)	0	19 (35)	1 (2)
Nausea	1 (100)	0	3 (60)	0	3 (50)	1 (17)	12 (33)	1 (3)	0	0	19 (35)	2 (4)
Infusion-related reaction	0	0	0	0	3 (50)	0	13 (36)	0	1 (17)	0	17 (31)	0
Vomiting	1 (100)	0	1 (20)	0	2 (33)	1 (17)	10 (28)	2 (6)	2 (33)	0	16 (30)	3 (6)
Constipation	1 (100)	0	0	0	3 (50)	0	10 (28)	0	1 (17)	0	15 (28)	0
Dyspnea	0	0	1 (20)	0	1 (17)	1 (17)	10 (28)	2 (6)	3 (50)	1 (17)	15 (28)	4 (7)
Anemia	0	0	1 (20)	0	3 (50)	1 (17)	10 (28)	6 (17)	0	0	14 (26)	7 (13)
Hypomagnesemia	0	0	3 (60)	0	5 (50)	0	7 (19)	0	1 (17)	0	14 (26)	0
Hypokalemia	1 (100)	0	2 (40)	0	0	0	9 (25)	4 (11)	1 (17)	0	13 (24)	4 (7)
Chills	0	0	0	0	2 (33)	0	11 (31)	0	0	0	13 (24)	0
Cough	0	0	1 (20)	0	1 (17)	0	8 (22)	0	1 (17)	0	11 (20)	0
Headache	0	0	2 (40)	0	1 (17)	0	7 (19)	0	0	0	10 (19)	0
Stomatitis	0	0	0	0	2 (33)	0	7 (19)	0	1 (17)	0	10 (19)	0
AST increased	0	0	2 (40)	0	1 (17)	0	6 (17)	0	0	0	9 (17)	0
Asthenia	0	0	0	0	2 (33)	1 (17)	5 (14)	1 (3)	2 (33)	1 (17)	9 (17)	3 (6)
Myalgia	0	0	0	0	1 (17)	0	7 (19)	0	1 (17)	0	9 (17)	0
Blood ALP increased	1 (100)	0	1 (20)	0	2 (33)	0	4 (11)	2 (6)	0	0	8 (15)	2 (4)
Hypophosphatemia	0	0	0	0	0	0	8 (22)	5 (14)	0	0	8 (15)	5 (9)
Pruritus	0	0	1 (20)	0	1 (17)	0	5 (14)	0	1 (17)	0	8 (15)	0
Pneumonia	0	0	0	0	0	0	8 (22)	6 (17)	0	0	8 (15)	6 (11)
Rash	0	0	0	0	1 (17)	0	6 (17)	0	1 (17)	0	8 (15)	0
Abdominal pain	0	0	0	0	0	0	6 (17)	1 (3)	1 (17)	0	7 (13)	1 (2)
Dehydration	0	0	1 (20)	0	2 (33)	1 (17)	4 (11)	2 (6)	0	0	7 (13)	3 (6)
Dry skin	0	0	1 (20)	0	2 (33)	0	4 (11)	0	0	0	7 (13)	0
Hypoalbuminemia	0	0	0	0	3 (50)	0	4 (11)	0	0	0	7 (13)	0
Peripheral edema	0	0	1 (20)	0	1 (17)	0	4 (11)	0	1 (17)	0	7 (13)	0
Weight decreased	0	0	1 (20)	0	2 (33)	0	4 (11)	0	0	0	7 (13)	0
Pleural effusion	0	0	1 (20)	1 (20)	0	0	4 (11)	1 (3)	1 (17)	1 (17)	6 (11)	3 (6)
Muscular weakness	0	0	1 (20)	0	1 (17)	0	2 (6)	0	2 (33)	0	6 (11)	0

*ALP* alkaline phosphatase, *AST* aspartate aminotransferase, *Q2W* once every two weeks, *QW* once weekly, *RDE* recommended dose for expansion

A total of 32 (59%) patients reported at least one SAE regardless of study drug relationship. The most frequently reported SAEs were pneumonia (13%), dehydration, dysphagia, dyspnea, and vomiting (6% each). A total of three patients (6%) reported SAEs suspected to be study drug-related (diarrhea plus hypokalemia, pneumatosis intestinalis, and pyrexia, in one patient each in the RDE treatment group). Seven on-treatment deaths were reported during the study, all in the RDE dose group; none were regarded as treatment-related. Two patients (4%) had AEs that led to study drug discontinuation: pneumonia (grade 3) and cerebrovascular accident (grade 2) in one patient, and increased alanine aminotransferase (ALT; grade 2), and increased aspartate aminotransferase (AST; grade 1), in the other – none were suspected to be study drug related. Overall, 32 patients (59%) reported AEs requiring dose adjustment or interruption (see Additional file 3: Table S3).

### Efficacy

**Fig. 1:**
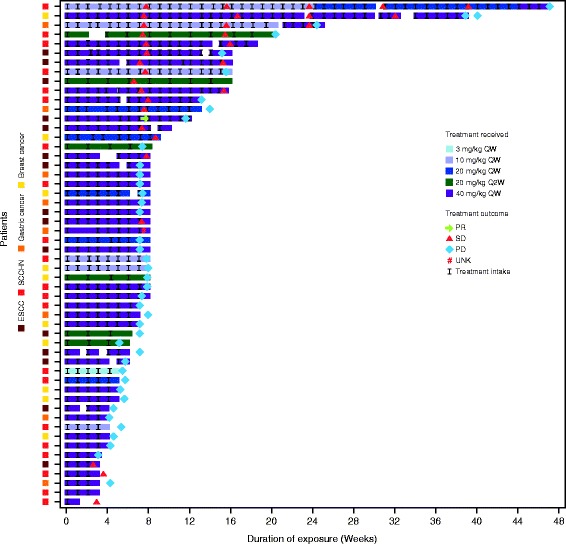
Duration of exposure and RECIST evaluation (FAS, *N* = 54). Footnote: *ESCC* esophageal squamous cell carcinoma, *PD* progressive disease, *PR* partial response, *Q2W* once every two weeks, *QW* once weekly, *RECIST* Response Evaluation Criteria In Solid Tumors, *SCCHN* squamous cell carcinoma of the head and neck, *SD* stable disease, *UNK* unknown

**Fig. 2:**
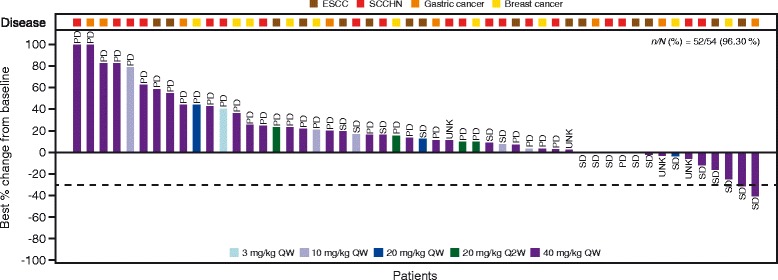
Best percentage change from baseline in target lesions by treatment group and indication (FAS). Footnote: *ESCC* esophageal squamous cell carcinoma, *PD* progressive disease, *Q2W* once every two weeks, *QW* once weekly, *SCCHN* squamous cell carcinoma of the head and neck, *SD* stable disease, *UNK* unknown

Of all treated patients, 17 (31%) achieved stable disease (SD) as best response, including one patient with SCCHN who achieved a long-lasting SD >40 weeks, one with HER2-positive metastatic breast cancer who achieved SD for approximately 32 weeks, and one trastuzumab-naive gastric cancer patient with an unconfirmed partial response at Day 53 (Cycle 2 Day 25) who subsequently experienced disease progression at Day 81 (Cycle 3 Day 25). Duration of exposure and RECIST evaluations are shown in Fig. 1. There were no complete or confirmed partial responses; tumor shrinkage was observed in several patients (Fig. 2). The median PFS for patients treated at the LJM716 RDE of 40 mg/kg QW was estimated to be 1.64 months (95% CI: 1.64–1.81 months).

### Pharmacokinetic studies

The clinical pharmacokinetic parameters for LJM716 are provided in Table 3. The exposure of LJM716 increased in an approximately dose-proportional manner in the dose range 3–40 mg/kg QW. There was 2–3.5-fold accumulation at Cycle 3 (the expected steady state) after repeated weekly doses. The effective half-life was estimated to be 9–14 days. For the RDE dose of 40 mg/kg QW, mean C_max_ and AUC_last_ of Cycle 1 Day 1, the drug accumulation and effective half-life from the dose-escalation phase, were comparable with the observations in the dose-expansion phase**.** Based on unpublished data, the target concentration associated with efficacy in the most sensitive mouse model (Fadu) was 125 μg/mL and approximately 500 μg/mL in all other models investigated. Administration of LJM718 40 mg/kg QW is expected to achieve the average steady-state concentration of 500 μg/mL in most of the patients.

**Table 3:** Primary pharmacokinetic parameters for LJM716 3–40 mg/kg QW (Cycle 1 Day 1)

	3 mg/kg QW *n* = 1	10 mg/kg QW *n* = 5	20 mg/kg QW *n* = 6	40 mg/kg QW *n* = 6	40 mg/kg QW EX, *n* = 30	20 mg/kg Q2W *n* = 6
AUC_last_, h*μg/mLa	6158 (−)	21,302 (3731)	32,791 (9635)	74,637 (7182)	73,360 (18,819)	52,659 (23,302)
C_max_, μg/mL	65.2 (−)	204 (23.6)	369 (131)	839 (143)	706 (129)	408 (137)
T_max,_ h	2.18 (2.18–2.18)	4.00 (2.13–4.53)	3.88 (2.08–5.33)	4.34 (2.42–8.42)	3.79 (2.05–7.5)	3.25 (2.08–4.03)
T_last,_ h	168 (168–168)	170 (166–171)	167 (146–169)	167 (166–191)	166 (7.22–171)	334 (261–336)
C_last_, μg/mL	21.4 (−)	80.3 (18.4)	115.0 (28.6)	296.0 (66.0)	236.0 (79.7)	82.0 (46.6)

Mean values (standard deviation) provided, except for T_max_ and T_last_ which are median (range)

*AUC,* area under curve, *EX* expansion phase, *Q2W* once every two weeks, *QW* once weekly

### Biomarker studies

1. Pharmacodynamic biomarker analysis
  A decrease in p-HER3/t-HER3 after LJM716 treatment was observed in three out of five paired tumor biopsy samples (Table 4).
2. Exploratory biomarkers
  Baseline archival tumor biopsy *NRG1* gene expression levels as measured by RT-PCR, and according to indication and treatment group, are shown in the Supplementary Material (see Additional file 4: Figure S1A). No obvious relationship between baseline NRG1 expression and response could be seen based on graphical review of the data. Some lineage differences in *NRG1* expression were seen; squamous cancer types, such as SCCHN and ESCC, tend to have higher *NRG1* expression, indicating a ligand-driven HER3 activation in these tumor types. Interestingly, relatively high *NRG1* expression was also observed in a number of HER2+ gastric cancer tumors and one breast cancer tumor, suggesting that these tumors may exhibit both ligand-dependent and ligand-independent activation of HER3.
  NGS studies indicated PI3K pathway activation through *PIK3CA* mutation (11 patients) and *PIK3CA* gene amplification (six patients); changes in *PTEN* genes, either through functional mutation, copy number loss, or frame shift, were also detected in five patients (see Additional file 4: Figure S1B). It is noteworthy that tumor shrinkage of >20% was observed in 2 patients with confirmed *PIK3CA* mutation (Additional file 4: Figure S1B).

**Table 4:** Biomarker inhibition in paired tumor biopsies of individual patients treated with LJM716 40 mg/kg QW

Patient biopsy	Best response	t-HER3	p-HER3	HER3 ratio (P/T)	t-AKT	p-AKT	AKT ratio (P/T)
1	PD	−86% ↑	81% ↓	90% ↓	7% ↓	84% ↓	83% ↓
2	SD	ND	−157% ↑	ND	−249% ↑	−84% ↑	47% ↓
3	PD	ND	63% ↓	ND	ND	ND	ND
4	SD	25% ↓	ND	ND	−33% ↑	0%	25% ↓
5	SD	72% ↓	82% ↓	34% ↓	70% ↓	ND	ND
Median		25%	72% ↓	62% ↓	−13% ↑	0%	47% ↓

Percentages represent changes in the post-baseline sample against the baseline value

Positive and negative values represent biomarker inhibition (↓) and stimulation (↑), respectively

*HER3* human epidermal growth factor receptor 3, *ND* not determined, *PD* progressive disease, *p-AKT* phosphor-AKT, *p-HER3* phospho-HER3, *Q2W* once every two weeks, *QW* once weekly, *SD* stable disease, *t-AKT* total AKT, *t-HER3* total HER3

### Immunogenicity

A total of 54 patients were tested for the presence of anti-drug antibodies (ADAs) to LJM716 using an assay with high drug tolerance. No ADAs to LJM716 were detected in any samples tested.

## Conclusions

Intravenously administered LJM716 was well tolerated, with an acceptable and manageable safety profile; on-target toxicities were largely grade 1 or 2, with no obvious dose proportionality. LJM716 has dose-dependent pharmacokinetic exposure and an effective half-life between 9 and 14 days. The RDE was established at 40 mg/kg QW, which provided systemic drug levels above the minimum effective concentration established from mouse xenograft models. The pharmacodynamic biomarker data indicated that levels of p-HER3 and t-HER3 were reduced in paired tumor samples, although pharmacodynamic data are limited.

This was a small cohort study in pretreated patients that was not designed to establish efficacy; no confirmed responses were observed, although tumor shrinkage was seen in some patients. Several factors may potentially explain the limited antitumor activity observed under single-agent treatment with LJM716. Patients with HER2-driven tumors did not continue HER2-directed therapy under LJM716 treatment, and it has been recently shown that trastuzumab-pretreated patients with HER2-driven breast tumors derive more benefit from a dual anti-HER2/HER3 treatment strategy [20]. Similarly, patients with SCCHN might benefit from combining LJM716 with anti-EGFR therapy. Furthermore, although ESCC and SCCHN are considered to have frequent deregulation in HER2/HER3, we did not preselect patients with such aberrations, and patients with other downstream mutations or amplifications were not excluded (11 patients had *PIK3CA* mutation and six patients had *PIK3CA* gene amplification). The results of this study are consistent with previous phase I data for the monoclonal HER3 antibody patritumab (U3–1287) which provided some evidence of disease stabilization in patients with solid tumors [21], and which subsequently demonstrated encouraging efficacy in combination with erlotinib [22]. Similarly, RG7116 combined with cetuximab or erlotinib has demonstrated preliminary signs of clinical activity in patients with HER3-expressing tumors [23]. Future studies of LJM716 will also evaluate LJM716 in combination with other therapeutic agents, including the PI3K inhibitor alpelisib (BYL719) in patients with ESCC, and as part of the triple combination of LJM716, alpelisib, and trastuzumab in patients with HER2-overexpressing breast cancer, in which preliminary data have indicated antitumor activity in patients with *PIK3CA* mutations [24]. Further studies to establish the correlation of serum and tumor biomarkers with LJM716 antitumor activity may also be warranted.

## Additional files

Additional file 1: Table S1. Summary of criteria for dose-limiting toxicities (CTCAE version 4.03 grading). Footnote: *ALT* alanine aminotransferase, *AST* aspartate aminotransferase*, CTCAE* Common Terminology Criteria for Adverse Events, *DLT*s dose-limiting toxicities, *ULN* upper limit of normal. (DOCX 28 kb)

Additional file 2: Table S2. Adverse events (all grades [≥10%] and grades 3/4) suspected to be drug-related, by treatment group. Footnote: All reported grade 3/4 treatment-related adverse events were of grade 3 severity. Additional grade 3 treatment-related adverse events not shown above: pneumatosis intestinalis (*n* = 1) and lipase increased (*n* = 1) in the 40 mg/kg QW RDE group, and asthenia (*n* = 1) in the 20 mg/kg QW group. *Q2W* once every two weeks, *QW* once weekly, *RDE* recommended dose for expansion. (DOCX 17 kb)

Additional file 3: Table S3. Adverse events (all grades [≥5%]) requiring dose adjustment/interruption, regardless of causality, by treatment group. Footnote: *Q2W* once every two weeks, *QW* once weekly, *RDE* recommended dose for expansion. (DOCX 15 kb)

Additional file 4: Figure S1. **A** Baseline NRG1 level in archival tumor samples by treatment group and indication. **B** Best percentage change from baseline in the sum of lesion diameters by mutational status and treatment; one additional patient with *PIK3CA* amplification, and who had non-target lesions only, is not shown. Footnote: *ERRB3* v-erb-b2 erythroblastic leukemia viral oncogene homolog 3, *ESCC* esophageal squamous cell carcinoma, *NRG1* neuregulin 1, *PIK3CA amp* PIK3CA amplified, *PD* progressive disease, *PTEN* phosphatase and tensin homolog, *Q2W* once every two weeks, *QW* once weekly, *SCCHN* squamous cell carcinoma of the head and neck, *SD* stable disease, *UNK* unknown; ∆Cq normalized gene expression. (PDF 201 kb)

## Abbreviations

ADA: anti-drug antibodies
AE: adverse event
ALP: alkaline phosphatase
ALT: alanine aminotransferase
AST: aspartate aminotransferase
AUC: area under curve
BLRM: Bayesian logistic regression model
CEER: Collaborative Enzyme Enhanced Reactive
CI: confidence interval
CT: computed tomography
DLT: dose-limiting toxicities
ECOG: Eastern Cooperative Oncology Group
EGFR: epidermal growth factor receptor
ErbB: v-erb-b2 erythroblastic leukemia viral oncogene homolog
ESCC: esophageal squamous cell carcinoma
EWOC: escalation with overdose control
EX: expansion phase
FAS: full analysis set
HER2: human epidermal growth factor receptor 2
HER3: human epidermal growth factor receptor
IgG1: immunoglobulin G1
IHC: immunohistochemistry
IRR: infusion-related reactions
IV: intravenously
MRI: magnetic resonance imaging
MTD: maximum tolerated dose
ND: not determined
NGS: next-generation sequencing
NRG1: neuregulin 1
ORR: overall response rate
p-AKT: phosphor-AKT
PD: progressive disease
PFS: progression-free survival
p-HER3: phospho-human epidermal growth factor receptor 3
PI3K: phosphoinositide 3-kinase
*PIK3CA* amp: *PIK3CA* amplified
PIK3CA: phosphoinositide 3-kinase, catalytic subunit alpha
PR: partial response
PS: performance status
PTEN: phosphatase and tensin homolog
Q2W: once every two weeks
QW: once weekly
RDE: recommended dose for expansion
RECIST: Response Evaluation Criteria in Solid Tumors
RTK: receptor tyrosine kinase
RT-PCR: reverse transcription polymerase chain reaction
SAE: serious adverse event
SCCHN: squamous cell carcinoma of the head and neck
SD: stable disease
t-AKT: total AKT
t-HER3: total human epidermal growth factor receptor 3
UNK: unknown

## References

[1] Amin DN, Campbell MR, Moasser MM (2010). The role of HER3, the unpretentious member of the HER family, in cancer biology and cancer therapeutics. Semin Cell Dev Biol.

[2] Jura N, Shan Y, Cao X, Shaw DE, Kuriyan J (2009). Structural analysis of the catalytically inactive kinase domain of the human EGF receptor 3. Proc Natl Acad Sci U S A.

[3] Holbro T, Beerli RR, Maurer F, Koziczak M, Barbas CF, Hynes NE (2003). The ErbB2/ErbB3 heterodimer functions as an oncogenic unit: ErbB2 requires ErbB3 to drive breast tumor cell proliferation. Proc Natl Acad Sci U S A.

[4] Garner AP, Bialucha CU, Sprague ER, Garrett JT, Sheng Q, Li S (2013). An antibody that locks HER3 in the inactive conformation inhibits tumor growth driven by HER2 or neuregulin. Cancer Res.

[5] Ghosh R, Narasanna A, Wang SE, Liu S, Chakrabarty A, Balko JM (2011). Trastuzumab has preferential activity against breast cancers driven by HER2 homodimers. Cancer Res.

[6] Swain SM, Kim SB, Cortés J, Ro J, Semiglazov V, Campone M (2013). Pertuzumab, trastuzumab, and docetaxel for HER2-positive metastatic breast cancer (CLEOPATRA study): overall survival results from a randomised, double-blind, placebo-controlled, phase 3 study. Lancet Oncol.

[7] Kono K, Mimura K, Fujii H, Shabbir A, Yong WP, Jimmy SA (2012). Potential therapeutic significance of HER-family in esophageal squamous cell carcinoma. Ann Thorac Cardiovasc Surg.

[8] Nahta R (2012). Pharmacological strategies to overcome HER2 cross-talk and Trastuzumab resistance. Curr Med Chem.

[9] Bang YJ, Van Cutsem E, Feyereislova A, Chung HC, Shen L, Sawaki A (2010). Trastuzumab in combination with chemotherapy versus chemotherapy alone for treatment of HER2-positive advanced gastric or gastro-oesophageal junction cancer (ToGA): a phase 3, open-label, randomised controlled trial. Lancet.

[10] Wilson TR, Lee DY, Berry L, Shames DS, Settleman J (2011). Neuregulin-1-mediated autocrine signaling underlies sensitivity to HER2 kinase inhibitors in a subset of human cancers. Cancer Cell.

[11] Meetze K, Vincent S, Tyler S, Mazsa EK, Delpero AR, Bottega S (2015). Neuregulin 1 expression is a predictive biomarker for response to AV-203, an ERBB3 inhibitory antibody, in human tumor models. Clin Cancer Res.

[12] Sheng Q, Pinzon-Ortiz M, Das R, Huang A, Rong X, Cao ZA. Targeting HER3 and IGF1R in NRG1 high lung squamous cell carcinoma. Cancer Res. 2014;74 Abstr. LB-237

[13] Wolff AC, Hammond ME, Hicks DG, Dowsett M, McShane LM, Allison KH (2013). Recommendations for human epidermal growth factor receptor 2 testing in breast cancer: American Society of Clinical Oncology/College of American Pathologists clinical practice guideline update. J Clin Oncol.

[14] Wolff AC, Hammond ME, Schwartz JN, Hagerty KL, Allred DC, Cote RJ (2007). American Society of Clinical Oncology/College of American Pathologists guideline recommendations for human epidermal growth factor receptor 2 testing in breast cancer. Arch Pathol Lab Med.

[15] Eisenhauer EA, Therasse P, Bogaerts J, Schwartz LH, Sargent D, Ford R (2009). New response evaluation criteria in solid tumours: Revised RECIST guideline (version 1.1). Eur J Cancer.

[16] Rogatko A, Schoeneck D, Jonas W, Tighiouart M, Khuri FR, Porter A (2007). Translation of innovative designs into phase I trials. J Clin Oncol.

[17] Babb J, Rogatko A, Zacks S (1998). Cancer phase I clinical trials: efficient dose escalation with overdose control. Stat Med.

[18] Neuenschwander B, Branson M, Gsponer T (2008). Critical aspects of the Bayesian approach to phase I cancer trials. Stat Med.

[19] U. S. Department of Health and Human Services (National Institutes of Health/National Cancer Institute). Common Terminology Criteria for Adverse Events (CTCAE) Version 4.03; June 14, 2010.

[20] Cortés J, Fumoleau P, Bianchi GV, Petrella TM, Gelmon K, Pivot X (2012). Pertuzumab monotherapy after trastuzumab-based treatment and subsequent reintroduction of trastuzumab: activity and tolerability in patients with advanced human epidermal growth factor receptor 2-positive breast cancer. J Clin Oncol.

[21] LoRusso P, Janne PA, Oliveira M, Rizvi N, Malburg L, Keedy V (2013). Phase I study of U3-1287, a fully human anti-HER3 monoclonal antibody, in patients with advanced solid tumors. Clin Cancer Res.

[22] Nishio M, Horiike A, Murakami H, Yamamoto N, Kaneda H, Nakagawa K (2015). Phase I study of the HER3-targeted antibody patritumab (U3-1287) combined with erlotinib in Japanese patients with non-small cell lung cancer. Lung Cancer.

[23] Lassen UN, Cervantes Ruiperez A, Fleitas T, Meulendijks D, Schellens J, Lolkemar M, et al. Phase IB trial of RG7116, a glycoengineered monoclonal antibody targeting HER3, in combination with cetuximab or erlotinib in patients with advanced/metastatic tumors of epithelial cell origin expressing HER3 protein. Ann Oncol. 2014;25(Suppl 4):Abstr. iv147:444O.

[24] Shah DP, Chandarlapaty S, Dickler MN, Ulaner G, Zamora SJ, Sterlin V, et al. Phase I study of LJM716, BYL719, and trastuzumab in patients (pts) with HER2-amplified (HER2+) metastatic breast cancer (MBC). J Clin Oncol. 2015;33(Suppl):Abstr. 590.

