# Supplementary material for: A phase I open-label dose-escalation study of the anti-HER3 monoclonal antibody LJM716 in patients with advanced squamous cell carcinoma of the esophagus or head and neck and HER2-overexpressing breast or gastric cancer
Source: BMC Cancer. 2017 Sep 12;17:646. doi: 10.1186/s12885-017-3641-6 (PMC5596462; doi:10.1186/s12885-017-3641-6)
Supplement: Supplementary file 1 — Summary of criteria for dose-limiting toxicities (CTCAE version 4.03 grading). Footnote: ALT alanine aminotransferase, AST aspartate aminotransferase, CTCAE Common Terminology Criteria for Adverse Events, DLTs dose-limiting toxicities, ULN upper limit of normal. (DOCX 28 kb) [file 12885_2017_3641_MOESM1_ESM.docx]

**Table S1** Summary of criteria for dose-limiting toxicities (CTCAE version 4.03 grading)

| DLTs are specified for various organs not limited to hematology, renal, hepatic, cardiac, and pulmonary |
| --- |
| Hematologic  ≥ grade 3 neutropenia for >7 consecutive days, ≥ grade 3 (with clinical bleeding) or grade 4 thrombocytopenia, grade 4 anemia, febrile neutropenia |
| Laboratory renal/hepatic  Serum creatinine >2 x ULN; ≥ grade 3 total bilirubin increased (or grade 2 with grade 3 AST/ALT increased), grade 4 AST/ALT increased (or grade 3 for >7 consecutive days if accompanied by liver metastases) |
| Cardiac and pulmonary  ≥ grade 2 heart failure or ≥ grade 3 cardiac event; ≥ grade 3 (or grade 2 lasting >7 days in same cycle) pulmonary event |
| Non-hematologic  Other ≥ grade 3 non-hematologic toxicity or investigator-assessed unacceptable toxicity considered to be dose limiting |
| Exceptions  <72 hours of CTCAE grade 3 fatigue; alopecia, inadequately treated nausea, vomiting, or diarrhea; clinically non-significant, treatable, or reversible laboratory abnormalities |

*ALT* alanine aminotransferase, *AST* aspartate aminotransferase*, CTCAE* Common Terminology Criteria for Adverse Events, *DLT*s dose-limiting toxicities, *ULN* upper limit of normal.
