# Supplementary material for: A phase I open-label dose-escalation study of the anti-HER3 monoclonal antibody LJM716 in patients with advanced squamous cell carcinoma of the esophagus or head and neck and HER2-overexpressing breast or gastric cancer
Source: BMC Cancer. 2017 Sep 12;17:646. doi: 10.1186/s12885-017-3641-6 (PMC5596462; doi:10.1186/s12885-017-3641-6)
Supplement: Supplementary file 2 — Adverse events (all grades [≥10%] and grades 3/4) suspected to be drug-related, by treatment group. Footnote: All reported grade 3/4 treatment-related adverse events were of grade 3 severity. Additional grade 3 treatment-related adverse events not shown above: pneumatosis intestinalis (n = 1) and lipase increased (n = 1) in the 40 mg/kg QW RDE group, and asthenia (n = 1) in the 20 mg/kg QW group. Q2W once every two weeks, QW once weekly, RDE recommended dose for expansion. (DOCX 17 kb) [file 12885_2017_3641_MOESM2_ESM.docx]

**Table S2** Adverse events (all grades [≥10 %] and grades 3/4) suspected to be drug-related, by treatment group

| Preferred term, *n* (%) | 3 mg/kg QW  *n* = 1 | | 10 mg/kg QW  *n* = 5 | | 20 mg/kg QW  *n* = 6 | | 40 mg/kg QW  RDE, *n* = 36 | | 20 mg/kg Q2W  *n* = 6 | | All patients  *N* = 54 | |
| --- | --- | --- | --- | --- | --- | --- | --- | --- | --- | --- | --- | --- |
|  | All grades | Grade 3/4 | All grades | Grade 3/4 | All grades | Grade 3/4 | All grades | Grade 3/4 | All grades | Grade 3/4 | All grades | Grade 3/4 |
| Diarrhea | 0 | 0 | 2 (40) | 0 | 3 (50) | 1 (17) | 15 (42) | 1 (3) | 3 (50) | 0 | 21 (39) | 2 (4) |
| Infusion-related reaction | 0 | 0 | 0 | 0 | 3 (50) | 0 | 13 (36) | 0 | 1 (17) | 0 | 17 (31) | 0 |
| Chills | 0 | 0 | 0 | 0 | 2 (33) | 0 | 11 (31) | 0 | 0 | 0 | 13 (24) | 0 |
| Decreased appetite | 0 | 0 | 1 (20) | 0 | 2 (33) | 0 | 9 (25) | 0 | 0 | 0 | 12 (22) | 0 |
| Pyrexia | 0 | 0 | 1 (20) | 0 | 0 | 0 | 10 (28) | 0 | 0 | 0 | 11 (20) | 0 |
| Hypomagnesemia | 0 | 0 | 3 (60) | 0 | 2 (33) | 0 | 5 (14) | 0 | 0 | 0 | 10 (19) | 0 |
| Vomiting | 1 (100) | 0 | 0 | 0 | 2 (33) | 0 | 6 (17) | 0 | 0 | 0 | 9 (17) | 0 |
| Fatigue | 0 | 0 | 2 (40) | 0 | 0 | 0 | 7 (19) | 0 | 0 | 0 | 9 (17) | 0 |
| Nausea | 1 (100) | 0 | 0 | 0 | 1 (17) | 0 | 6 (17) | 0 | 0 | 0 | 8 (15) | 0 |
| Hypokalemia | 1 (100) | 0 | 1 (20) | 0 | 0 | 0 | 5 (14) | 1 (3) | 0 | 0 | 7 (13) | 1 (2) |
| Stomatitis | 0 | 0 | 0 | 0 | 1 (17) | 0 | 4 (11) | 0 | 1 (17) | 0 | 6 (11) | 0 |

All reported grade 3/4 treatment-related adverse events were of grade 3 severity.

Additional grade 3 treatment-related adverse events not shown above: pneumatosis intestinalis (*n* = 1) and lipase increased (*n* = 1) in the
40 mg/kg QW RDE group, and asthenia (*n* = 1) in the 20 mg/kg QW group.

*Q2W* once every two weeks, *QW* once weekly, *RDE* recommended dose for expansion.
