# Supplementary material for: A phase I open-label dose-escalation study of the anti-HER3 monoclonal antibody LJM716 in patients with advanced squamous cell carcinoma of the esophagus or head and neck and HER2-overexpressing breast or gastric cancer
Source: BMC Cancer. 2017 Sep 12;17:646. doi: 10.1186/s12885-017-3641-6 (PMC5596462; doi:10.1186/s12885-017-3641-6)
Supplement: Supplementary file 3 — Adverse events (all grades [≥5%]) requiring dose adjustment/interruption, regardless of causality, by treatment group. Footnote: Q2W once every two weeks, QW once weekly, RDE recommended dose for expansion. (DOCX 15 kb) [file 12885_2017_3641_MOESM3_ESM.docx]

**Table S3** Adverse events (all grades [≥5%]) requiring dose adjustment/interruption, regardless of causality, by treatment group

| Preferred term, *n* (%) | 3 mg/kg QW  *n* = 1 | 10 mg/kg QW  *n* = 5 | 20 mg/kg QW  *n* = 6 | 40 mg/kg QW  RDE, *n* = 36 | 20 mg/kg Q2W  *n* = 6 | All patients  *N* = 54 |
| --- | --- | --- | --- | --- | --- | --- |
| Infusion-related reaction | 0 | 0 | 3 (50) | 9 (25) | 0 | 12 (22) |
| Chills | 0 | 0 | 2 (33) | 8 (22) | 0 | 10 (19) |
| Pneumonia | 0 | 0 | 0 | 4 (11) | 0 | 4 (7) |
| Hypercalcemia | 0 | 0 | 0 | 3 (8) | 0 | 3 (6) |
| Hypophosphatemia | 0 | 0 | 0 | 3 (8) | 0 | 3 (6) |
| Tremor | 0 | 0 | 1 (17) | 2 (6) | 0 | 3 (6) |
| Vomiting | 0 | 0 | 1 (17) | 2 (6) | 0 | 3 (6) |

*Q2W* once every two weeks, *QW* once weekly, *RDE* recommended dose for expansion.
