# Supplementary figures and images for: A phase I open-label dose-escalation study of the anti-HER3 monoclonal antibody LJM716 in patients with advanced squamous cell carcinoma of the esophagus or head and neck and HER2-overexpressing breast or gastric cancer
Source: BMC Cancer. 2017 Sep 12;17:646. doi: 10.1186/s12885-017-3641-6 (PMC5596462; doi:10.1186/s12885-017-3641-6)

**A**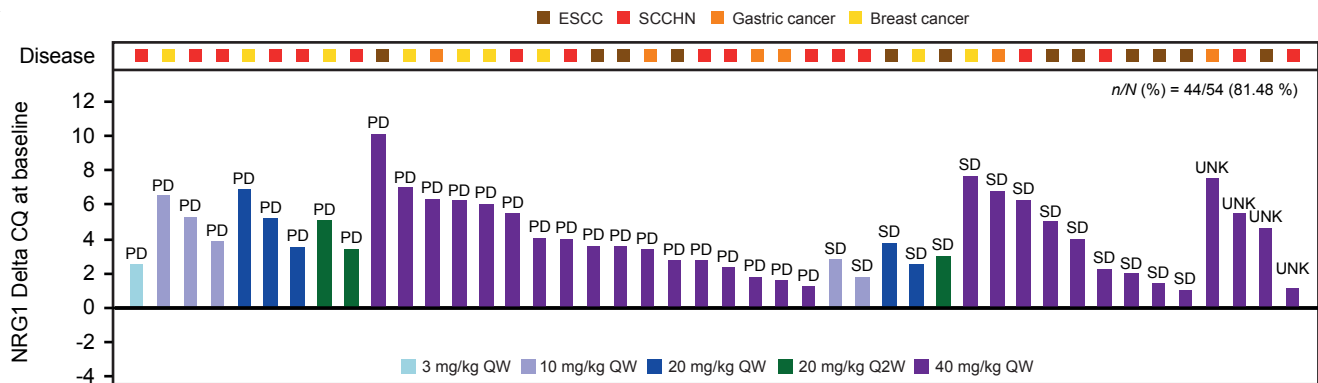**B**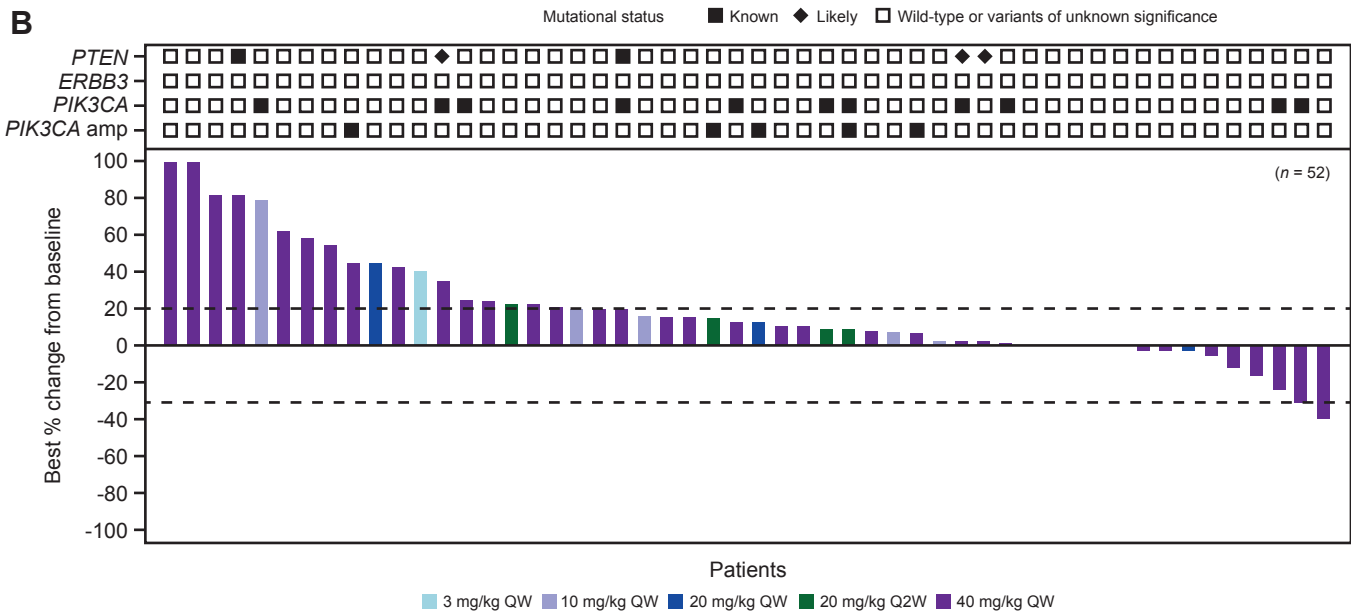

Supplement: Supplementary file 4 — A Baseline NRG1 level in archival tumor samples by treatment group and indication. B Best percentage change from baseline in the sum of lesion diameters by mutational status and treatment; one additional patient with PIK3CA amplification, and who had non-target lesions only, is not shown. Footnote: ERRB3 v-erb-b2 erythroblastic leukemia viral oncogene homolog 3, ESCC esophageal squamous cell carcinoma, NRG1 neuregulin 1, PIK3CA amp PIK3CA amplified, PD progressive disease, PTEN phosphatase and tensin homolog, Q2W once every two weeks, QW once weekly, SCCHN squamous cell carcinoma of the head and neck, SD stable disease, UNK unknown; ∆Cq normalized gene expression. (PDF 201 kb) [file 12885_2017_3641_MOESM4_ESM.pdf]
